# Tumor-infiltrating lymphocytes and CD8^+^ T cells predict survival of triple-negative breast cancer

**DOI:** 10.1007/s00432-019-03036-5

**Published:** 2019-09-27

**Authors:** H. Vihervuori, T. A. Autere, H. Repo, S. Kurki, L. Kallio, M. M. Lintunen, K. Talvinen, P. Kronqvist

**Affiliations:** 1 Department of Pathology, Institute of Biomedicine, University of Turku, Turku, Finland; 2 Auria Biobank, Turku, Finland; 3 Department of Pathology, Turku University Hospital, Turku, Finland

**Keywords:** TNBC, Inflammation, Proliferation, TIL, CD8, Prognosis

## Abstract

**Purpose:**

Tumor inflammatory response was evaluated as a prognostic feature in triple-negative breast cancer (TNBC) and compared with the clinical prognosticators of breast cancer and selected biomarkers of cancer cell proliferation.

**Methods:**

TNBC patients (*n* = 179) with complete clinical data and up to 18-year follow-up were obtained from Auria biobank, Turku University Hospital, Turku, Finland. Tumor-infiltrating lymphocytes (TILs) and several subtypes of inflammatory cells detected with immunohistochemistry were evaluated in different tumor compartments in full tissue sections and tissue microarrays.

**Results:**

Deficiency of stromal TILs and low number of CD8^+^ T cells independently predicted mortality in TNBC (HR 2.4, *p* 0.02 and HR 2.1, *p* 0.02, respectively). Each 10% decrease in stromal TILs resulted in 20% increased risk of mortality. An average of 13.2-year survival difference was observed between the majority (> 75%) of patients with low (< 14% of TILs) vs high (≥ 14% of TILs) frequency of CD8^+^ T cells. The prognostic value of TILs and CD8^+^ T cells varied when evaluated in different tumor compartments. TILs and CD8^+^ T cells were significantly associated with Securin and Separase, essential regulators of metaphase–anaphase transition of the cell cycle.

**Discussion:**

TILs and CD8^+^ T cells provide additional prognostic value to the established clinical prognostic markers in TNBC. However, possible clinical applications would still benefit from systematic guidelines for evaluating tumor inflammatory response. Increasing understanding on the interactions between the regulation of cancer cell proliferation and inflammatory response may in future advance treatment of TNBC.

## Introduction

Tumor microenvironment—the combination of neoplastic, inflammatory and pro-tumoral stromal cells and their associated soluble factors—conducts cellular interactions with crucial roles in malignancy (Monnot and Romero 2018). Of particular interest is the inflammatory cell component which, depending on the immunogeneity of the neoplasm, may be involved in complicated tumor-promoting or -suppressing mechanisms. Among these, inflammatory cells may either suppress tumor growth through destruction of malignant cells or, conversely, establish an immunosuppressive microenvironment which favors escape of the tumor cells from the anti-tumoral immune response (da Silva et al. 2019). Tumor microenvironment is also known to enhance tumor progression by recruiting stromal cells to provide growth signals stimulating cell proliferation and metastatic capacity. Understanding the crosstalk between immune response and proliferative activity may provide potential new prognostic and predictive markers for cancer (Yuan et al. 2016).

Reflecting the versatile involvement of immune response in malignancy, inflammatory cells have been reported with numerous and partly discrepant roles in different types of tumors. Recently, specific subtypes of inflammatory cells and their impact on disease survival and treatment response have been described in different subtypes of breast cancer (Yang et al. 2018). Among these, triple-negative breast carcinoma (TNBC) and, particularly its so-called immunomodulatory subtype, have been reported with immunogenic properties distinct from other breast carcinomas (Matsumoto et al. 2015; Lehmann et al. 2011).

TNBC commonly affects younger women, and is known for particularly aggressive clinical behavior and sinister outcome (Bianchini et al. 2016). For long, TNBC has comprised a specific treatment challenge due to lack of targeted therapeutic options. Recently, however, immune checkpoint-based therapies exploiting the immune/tumor interaction have provided for PD-L1^+^ metastatic or locally advanced TNBC significant survival benefit (Schmid et al. 2018). There are hopes that gaining more understanding on tumor-infiltrating lymphocytes (TILs) and the prevalence of the TIL subpopulations might reveal novel biomarkers for TNBC. Also, the interrelation between cell proliferation and tumor microenvironment has been suggested with prognostic and predictive potential in cancer (Haschka et al. 2018). Particularly, abnormal proliferation resulting in chromosomal instability (CIN) and aneuploid DNA content—common features of TNBC—have been observed in association with upregulated expression of genes mediating immune response based on stimulation of pro-inflammatory signals (Santaguida et al. 2017).

In this study, specific features of inflammatory response are characterized in TNBC. The study is based on a total of 179 patients with complete clinical data and up to 18-year follow-up. Among the studied markers of inflammatory response and the established clinicopathological risk factors of breast cancer, only TILs and CD8^+^ cytotoxic T cells were significantly associated with disease-specific survival in TNBC. Previous literature has suggested that overexpession of the metaphase–anaphase regulators Securin and Separase promote cell proliferation and CIN in cancer and predict significantly increased breast cancer mortality (Nasmyth 2002; Gurvits et al. 2017). In the present findings, we also observed an association between increased immunoexpression of Securin and Separase and decreased prevalence of TILs and CD8^+^ T cells supporting previous hypotheses that dysfunctional cell proliferation may be interrelated to inflammatory reaction in TNBC.

## Materials and methods

### Patients

The study comprises 179 women diagnosed with unilateral TNBC in Turku University Hospital, Turku, Finland, during 2000–2015 (Table 1). The cases were included in the material based on WHO criteria (Lakhani et al. 2012) and St. Gallen consensus guidelines for surrogate markers of molecular subclassification (Coates et al. 2015). All patients were surgically treated with resection or mastectomy. Following resection, the patients were submitted for radiation therapy. The use of cytostatic drugs was based on international guidelines for treatment of TNBC at the time of diagnosis (Goldhirsch et al. 2009). None of the patients received neoadjuvant treatment. Complete clinical and follow-up data were collected from patient files available through Auria biobank, Turku University Hospital, Turku, Finland (http://www.auriabiobank.fi). Causes of death were obtained from autopsy reports, death certificates and from Finnish Cancer Registry (http://www.cancer.fi) resulting in maximum follow-up period of 18 years (mean 8 years).

**Table 1:** Summary of the clinical features of the patients (*n* = 179)

Mean age at diagnosis (range) (years)	60 (27–92)
Axillary lymph node metastasis (%)	33
Mean tumor size (range) (cm)	2.5 (0.5–8.5)
Basal differentiation (%)	83
Mean Ki-67 (range) (%)	50.2 (2–90)
Breast cancer deaths (%)	29

### Tissues

Formalin-fixed (pH 7.0) and paraffin-embedded archival tumor tissue of each patient was available through Auria biobank. The most representative tumor block of each patient was selected by experienced breast pathologists (HR and PK) and was available for the study as full tumor section and in tissue microarray (TMA). The full sections allowed evaluation of the central and peripheral areas of the tumors, as well as non-tumorous tissue outside the tumor borders. The TMAs comprised two tissue cores of each tumor, one from the central and another from the peripheral area. The TMAs were prepared by first identifying representative tumor areas on scanned images of HE-stained sections (3D HISTOTECH, Budapest, Hungary), then punching 1.5-mm-diameter cylinders from the blocks and, finally, constructing the tissue cores into TMAs using an automated tissue arrayer (TMA Grand Master machine, 3D HISTOTECH, Budapest, Hungary).

### Immunohistochemistry (IHC)

Inflammatory cells expressing CD8, CD20 and CD68 were detected by immunohistochemistry using BenchMark XT immunostainer and CD163, FoxP3 and MAC387 clone to recognize S100A8/9/12 complex expressed by macrophages and monocytes using Discovery XT (Roche Diagnostics/Ventana Medical Systems, Tucson, AZ, USA) following the standard immunohistochemical staining procedures of a pathology laboratory (Table 2). Securin and Separase were detected using Labvision Autostainer (Thermo-Fisher Scientific, Fremont, CA, USA) as described earlier (Gurvits et al. 2017). Expressions for estrogen (ER) and progesterone receptors (PR), as well as HER2 amplification, were ruled out using standard IHC practice and, in case of Her2-immunopositivity scores 2+ and 3+, by negative HER2 amplification status in double in situ hybridizations with chromosome 17 probe (Wolff et al. 2014; Goldhirsch et al. 2013). Expressions for epidermal growth factor receptor (EGFR) and cytokeratins 5 and 6 (CK5/6) were detected according to standard IHC practice and used to indicate basal differentiation (Lakhani et al. 2012).

**Table 2:** Summary of used antibodies

	Origin	Clone	Source	Dilution
CD8	Rabbit	SP57	Roche Diagnostics/Ventana	RTU
CD20	Mouse	L26	Roche Diagnostics/Ventana	RTU
CD68	Mouse	PG-M1	Dako	1:100
CD163	Rabbit	K20-T	Novus Biologicals	1:50
FoxP3	Mouse	236A/E7	Abcam	1:100
S100A8/A9	Mouse	MAC387	Novus Biologicals	1:50
Securin	Mouse	DCS-280	Novus Biologicals	1:100
Separase	Mouse	6H6	Abnova	1:1000
ER	Rabbit	SP1	Roche Diagnostics/Ventana	RTU
PR	Rabbit	1E2	Roche Diagnostics/Ventana	RTU
Her2	Rabbit	4B5	Roche Diagnostics/Ventana	RTU
Ki-67	Rabbit	30-9	Roche Diagnostics/Ventana	RTU
EGFR	Rabbit	5B7	Roche Diagnostics/Ventana	RTU
CK5/6	Mouse	D5 and 16B4	Roche Diagnostics/Ventana	RTU

### Evaluation of tumor-infiltrating lymphocytes (TILs)

Morphological evaluation of TILs was performed by an experienced breast pathologist (PK) in digitalized images of full tissue sections (4 µm, magnification 400×) comprising the whole tumor or at least a representative 15-mm-diameter area of the infiltrative tumor. The inflammatory response was defined as infiltration of mononuclear cells, excluding polymorphonuclear leukocytes from the analyses. All evaluations were performed avoiding areas with necrosis, suboptimal preservation, previous biopsy site and technical artifacts. The evaluations were performed following the international consensus recommendations (Salgado et al. 2015; Hendry et al. 2017; https://www.tilsinbreastcancer.org/) and the known biologically and clinically relevant morphological patterns of inflammatory infiltrates in breast cancer (Salgado et al. 2015).

To begin with the evaluations, the extent of TILs was registered as the area fraction (%) of the total stromal component of the tumor (so-called stromal TIL). In addition to evaluating the whole tumor area, stromal TILs were evaluated separately in the central area and in the peripheral invasive front of the tumors. Next, the extent of TILs was evaluated in the malignant epithelial compartment by registering the inflammatory cells in cell-to-cell contact with cancer cells and determining their number in relation (%) to cancer cells as the average from 3 sets of 100 cancer cells (so-called intratumoral TIL). Also these evaluations were repeated independently in the whole tumor as well as in the central area and in the invasive front of the tumors. Finally, outside the tumor in the adjacent normal tissue, the existence of tertiary lymphoid structures (TLS) was registered. TLSs were defined as a lymphocyte aggregates with a distinguishable T-cell zone and a B-cell follicle and registered as present vs absent.

### Immunohistochemical evaluation of inflammatory response

Immunohistochemical evaluations were performed on the TMAs by quantifying the fraction of immunopositive inflammatory cells separately in tissue cores representing the central and peripheral areas of each tumor. IHC for CD8 and CD20 was evaluated using the automated image analysis software ImmunoRatio (HV) (version 1.0c) for ImageJ (version 1.51s) (Institute of Biomedical Technology, University of Tampere, Tampere, Finland) (Tuominen et al. 2010). To ensure concordance of evaluations throughout the material, the thresholds for registering immunopositivity were determined based on visual observation without using blank field correction and the initially set thresholds were applied throughout the material. IHC for CD68, CD163, FoxP3 and MAC387 clone was evaluated subjectively (PK). The fraction (%) of CD68 immunopositive cells was calculated in separate representative tumor foci in relation to sets of 100 cancer cells (minimum one and maximum three foci) and the average value of the foci was registered for each tissue core. Due to their diffuse staining patterns, CD163, FoxP3 and MAC387 clones were classified into negative vs positive subgroups. Immunoevaluations for Securin and Separase were performed as previously described (Gurvits et al. 2016, 2017). Tissue cores with suboptimal tissue preservation or less than 100 cancer cells were excluded from the analysis.

### Statistical analysis

In statistical analyses, TILs were evaluated as continuous variables since, in literature, no formal recommendations for clinically relevant thresholds TIL have been given this far (Salgado et al. 2015). Two categories (present vs absent) were used in the analysis of TLSs. Immunoexpressions for CD8, CD20 and CD68 were classified into subgroups with low vs high inflammatory response based on the median value calculated separately for the central and the peripheral tissue core of each tumor. Correspondingly, CD163, FoxP3 and Mac387 clone were analyzed in two categories (negative vs positive). IHC for Securin and Separase was categorized into subgroups with high vs low immunoexpression applying thresholds presented in the previous literature (Gurvits et al. 2016, 2017).

Clinical parameters, survival rates and each of the studied inflammatory markers were first analyzed using contingency tables and Fisher’s exact test detecting differences in frequencies. Prognostic explorations of the data were performed using Kaplan–Meier estimates to demonstrate the cumulative percentages of breast cancer-specific mortality. Log rank tests and Cox’s regression models were used to assess associations between disease outcome, extent of inflammatory response and clinical prognostic features, i.e., patient’s age at diagnosis, tumor size and axillary lymph node status. Each association between the studied proteins and the routine prognosticator was quantitated as hazard ratio (HR) with 95% confidence interval (CI). *p* values under 0.05 were considered significant. Patients with missing data were censored from the data. Statistical analyses were performed with R Statistical Software (R Development Core Team 2017). The survival analysis package (Therneau and Lumley 2019) was used for Cox regression models, while Kaplan–Meier plots were drawn using the Survminer package (Kassambara and Kosinski 2018).

## Results

As evaluated from the whole tissue sections, the average area fraction of stromal TILs in the TNBCs was 29.1% (SE 1.8%). Almost one-third (29.9%) of the tumors showed TILs in more than 50% of the stromal area and 5.0% in more than 90% of the stroma area (Table 3). In 11.7% of tumors no stromal TILs were observed. When evaluated from different tumor areas, TIL infiltration was encountered more commonly in the invasive front than in the central part of the tumors. Also, the average area fraction of stromal TIL infiltration was more extensive in the invasive front (32.8%) than in the central part (22.9%) of the tumor. The fraction of intratumoral TILs in relation to cancer cells was 4.8% (SE 0.5) as evaluated from the whole tumor area. Intratumoral inflammatory reaction in the tumors was sparse so that clear expression (> 10%) of intratumoral TILs was observed in 12.3% of cases while 38.1% of cases showed no intratumoral TILs. In our material, the presence of intratumoral TILs was associated with higher than average extent of stromal TILs (35.3%) while in the absence of intratumoral TILs also the extent of stromal TILs was decreased (15.3%). The extent of intratumoral TILs did not markedly differ between the central area (5.6%) and the invasive front (4.3%) of the tumor. Only a single tumor in the material was observed with tertiary lymphoid structures, possibly because the perimeter of the tumor was not abundantly represented in the sections. The area fraction of stromal TILs was also evaluated in association with features of malignant cell proliferation and CIN based on Securin and Separase IHC. In the results, low area fraction of stromal TILs was significantly associated with high immunoexpressions for Securin (≥ 10% of cancer cells) (*p* = 0.003) and Separase (≥ 1% of cancer cells) (*p* = 0.01).

**Table 3:** The fraction (%) of TNBC patients (*n* = 179) showing stromal and intratumoral TILs as evaluated from the whole tumor area, from tumor center and invasive front

	Stromal TILs (%)	Intratumoral TILs (%)
Whole tumor area	88.3	61.9
TIL > 10%	54.2	87.7
TIL > 20%	44.1	8.9
TIL > 50%	29.9	< 1
TIL > 90%	5.0	0
Tumor center	22.9	49.2
Invasive front	33.8	58.1

The table also shows the fractions of patients with tumors showing different extents (> 10%, > 20%, > 50% and > 90%) of stromal and intratumoral TIL infiltrations

Immunohistochemical expressions of CD8, CD20, CD68, CD163, FoxP3 and MAC387 clone were evaluated from the TMA cores representing the center and the invasive front of the tumors. CD8 immunopositivity was seen in almost all TNBCs and equally expressed in TMA cores from the central area and invasive front (Table 4). Among the CD8^+^ tumors, an average of 24.6% (range 0–83.6) of TILs was immunopositive as compared to all tumor infiltrating inflammatory cells. CD20 immunopositivity was observed in the central area as well as the invasive front in slightly more than half of the tumors and the fraction of CD20^+^ TILs in the immunopositive tumors was 9.2% (range 0–66.5). CD68 was also regularly observed in the TNBCs, in the central area as well as in the invasive front. The average fraction of CD68^+^ TILs in the immunopositive tumors was 4.8% (range 0–25). As evaluated from the central cores, CD163, FoxP3 and MAC387 clone immunopositivity was observed in 53.3%, 45.0% and 21.2% of the TNBCs, respectively. Evaluating from tissue cores representing the invasive front, immunopositivity for CD163 and FoxP3 and MAC387 clone was more frequent (71.4%, 51.4% and 38.7% of the TNBCs, respectively). Concerning cancer cell proliferation, low fraction of CD8^+^ TILs was associated with high immunoexpression of Securin (≥ 10% of cancer cells) (*p* = 0.02) but not with Separase-IHC.

**Table 4:** The fraction (%) of TNBC patients (*n* = 147) showing immunopositivity for CD8, CD20 and CD68 in the TMA cores representing tumor center and invasive front

	CD8	CD20	CD68
Tumor center			
IHC positive	77.5	60.0	70.6
> 10% of TILs	54.1	20.2	6.5
> 20% of TILs	33.3	5.8	0
> 50% of TILs	8.2	1.7	0
> 90% of TILs	0	0	0
Invasive front			
IHC positive	78.1	61.2	79.6
> 10% of TILs	53.5	12.3	4.3
> 20% of TILs	31.4	3.1	0
> 50% of TILs	8.8	0	0
> 90% of TILs	0	0	0

The table also shows the fractions of patients with tumors showing different extents (> 10%, > 50%, > 90%) of immunopositive TILs

**Fig. 1:**
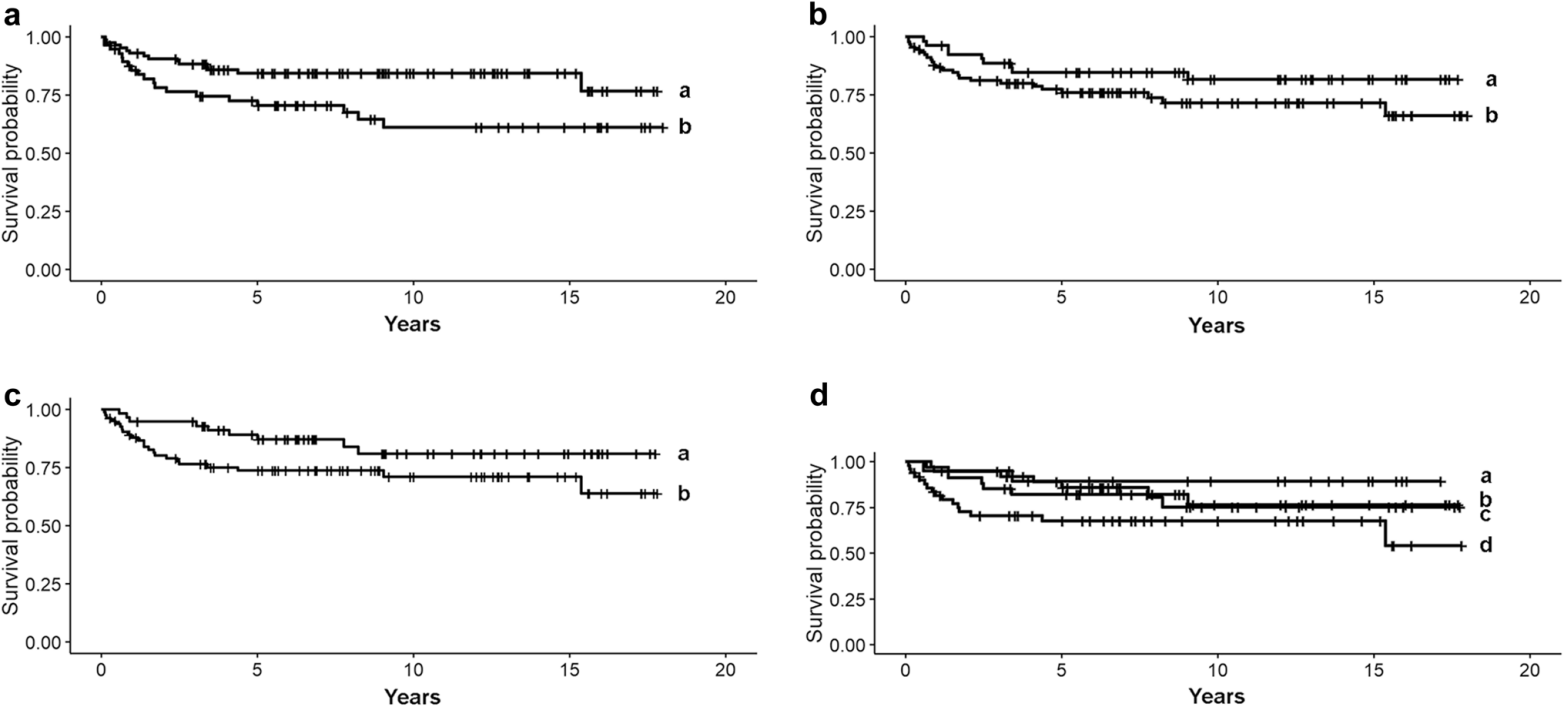
Survival of TNBC based on inflammatory response, tumor size and menopausal status of the patients (*n* = 179). **a** Patients showing high (≥ 14%, curve a) vs low fraction (< 14%, curve b) of CD8^+^ T cells (*p* = 0.02), **b** pre- (curve a) vs postmenopausal (curve b) patients (*p* = 0.03), **c** small (< 2 cm, curve a) vs large (≥ 2 cm, curve b) tumor size (*p* = 0.02), **d** small tumor size and premenopausal status (curve a) vs large tumor size and postmenopausal status (curve d) (*p* = 0.001). The survival of patients with small tumor size and postmenopausal status (curve b) does not significantly differ from patients with large tumor size and premenopausal status (curve c)

Among all studied indicators of inflammatory response, the density of TILs and CD8-IHC showed prognostic value in TNBC (Table 5). Concerning TILs, the area fraction of stromal TILs was associated with breast cancer-specific survival while the extent of intratumoral TILs or the presence of TLS did not predict disease outcome. High fraction of stromal TILs in the central area of the tumor—but not in the whole tumor area or in the invasive front—was significantly associated with favorable outcome of disease. Evaluated as a continuous variable, stromal TILs in the central area of the tumor predicted 2.4-fold increased probability of disease survival (*p* = 0.02). The practical interpretation is that each 10% increment in stromal TIL indicated 20% reduced risk of death in TNBC. More favorable outcome was also observed for TNBCs rich in CD8^+^ TILs. Instead, low frequency of CD8-positive inflammatory cells (< 14% of TILs), as evaluated from the TMA core representing the periphery of the tumor, predicted more than doubled risk of breast cancer death (HR 2.1, *p* = 0.02) (Fig. 1). The frequency of CD8^+^ TILs evaluated from the TMA core representing the tumor center sparsely failed to show statistical significance (*p* = 0.06). In our analyses, no statistically significant prognostic associations were observed for CD20, the studied macrophage markers or FoxP3.

**Table 5:** Univariate prognostic analyses involving TILs, immunohistochemical (IHC) inflammatory markers and established clinical prognosticators revealed the prognostic values of stromal TILs, CD8-IHC, tumor size and the patients’ menopausal status at diagnosis of TNBC (*n* = 147) individually and in combinations. The features with independent prognostic value were tested in multivariate analysis. The results are expessed as razard ratios (HR) and 95% confidence intervals (CI) of breast cancer-spesific mortality. Only assosiations with statistical signifigance (*p *< 0.05) are presented

	HR	*p*	CI
Univariate analysis			
TIL (as continuous variable)	2.4	0.02	1.2–5.2
CD8 (< 14% of TILs)	2.1	0.02	1.1–4.5
Tumor size (≥ 2 cm in diameter)	2.0	0.02	1.2–3.6
Postmenopausal status	2.2	0.03	1.2–4.2
TIL and tumor size	1.8	0.003	1.0–2.6
TIL and postmenopausal status	1.8	0.002	1.1–3.5
TIL and tumor size and postmenopausal status	2.0	0.005	1.1–3.9
CD8 and tumor size	2.9	0.004	1.1–3.8
Multivariate analysis model 1			
TIL	2.2	0.03	1.0–3.8
Tumor size	4.4	0.001	1.2–3.2
Postmenopausal status	2.9	0.004	1.1–3.0
Multivariate analysis model 2			
CD8	1.8	0.005	1.1–4.4
Tumor size	2.2	0.001	1.1–3.6

Among the studied clinical prognosticators, large tumor size (≥ 2 cm in diameter) and postmenopausal status at the time of diagnosis showed significantly decreased survival in TNBC (Table 5). An even stronger prognostic association was observed when comparing the subgroup of postmenopausal patients with large tumor size with the subgroup of premenopausal patients with small tumors (HR 2.9, *p* = 0.004). Also, the patient subgroups combining large tumor size, postmenopausal status or the combination of both and low area fraction of stromal TILs were associated with up to twofold risk of breast cancer mortality (*p* < 0.005). Correspondingly, patients with large tumors and low frequency of CD8^+^ TILs were associated with 2.9-fold increased risk of cancer mortality as compared to patients with small tumor size rich in CD8^+^ TILs (*p* = 0.001). No prognostic associations were observed for axillary lymph node status or basal differentiation of cancer cells.

In multivariate analyses (Table 5), low area fraction of stromal TILs and low frequency of CD8^+^ TILs were found to be independent prognosticators of survival in TNBC (HR 2.2, *p* 0.03 and HR 1.8, *p* 0.005, respectively), along with large tumor size and postmenopausal status.

## Discussion

TILs represent a vital component of the local anti-cancer immune response. In recent years, TILs have been proposed with prognostic value in several malignancies, including melanomas and carcinomas of the upper and lower gastrointestinal tract (Balatoni et al. 2018; Zheng et al. 2017; Galon et al. 2012). In breast cancer, the association of tumor-infiltrating lymphocytes with disease outcome has been recognized since decades (Moore and Foote 1949) and has, more recently, been verified in a number of large studies (Yu et al. 2016; Mao et al. 2016; Savas et al. 2016). Among breast carcinomas, TNBC comprises a distinct disease entity with a unique microenvironment of TILs and TAMs, and high proliferative activity with frequent CIN of the cancer cells (Yu and Di 2017; Yang et al. 2018). In our results, low area fraction of stromal TILs in the central area of the tumor predicted 2.4-fold increased probability of disease survival (*p* = 0.02). The practical interpretation of our results is that each 10% decrease in the fraction of stromal TILs results in 20% increased risk of mortality in TNBC, corresponding to findings in the previous literature (Loi et al. 2014). In multivariate analyses, deficiency of stromal TILs was an independent prognosticator of mortality in TNBC (HR 2.2, *p* = 0.03). Previously, corresponding conclusions on the associations between the frequency of TILs and outcome of TNBC have been presented both on the basis of morphological observations as well as expression profiling of immunomodulatory genes (Loi et al. 2014; Ibrahim et al. 2014; Desmedt et al. 2008). In the literature, TILs have even been suggested to predict the prognosis of residual disease after neoadjuvant treatment (Dieci et al. 2018; Denkert et al. 2015). In agreement with our results, tissue-assosiated macrophages have not been shown with independent prognostic value in TNBC (Mahmoud et al. 2012; Miyasato et al. 2017) although they have been suggested to promote proliferative activity, tumor growth and disease progression (Santoni et al. 2018; Levano et al. 2011).

In previous literature, divergent findings have been presented on the value of TIL subgroups in predicting the outcome of different malignancies. Prognostic associations have most commonly been observed for CD8^+^ cytotoxic T cells, but in some malignancies also for FOXP3^+^ regulatory and CD4^+^ helper T cells (de la Cruz-Merino et al. 2013). Activated CD8^+^ T lymphocytes are critically involved in the adaptive immunological defense and are known to kill cancer cells by several mechanisms (Martínez-Lostao et al. 2015). In our results, low frequency of CD8^+^ inflammatory cells (< 14% of TILs) in the periphery of the tumor predicted 2.1-fold increased risk of mortality in TNBC (*p* = 0.02). Concluding from the Kaplan–Meier curves (Fig. 1), the majority (75%) of patients with decreased fraction of CD8^+^ TILs (< 14% of TILs) died within an average of 2.2 years after diagnosis whereas the majority of patients with high frequency (≥ 14% of TILs) were alive in average 15.4 years after diagnosis. The observed association between low frequency of CD8^+^ TILs and unfavorable outcome of TNBC is in line with the main part of the literature (Ibrahim et al. 2014; Ali et al. 2014) although others have reported a reversed association between CD8^+^ TILs and disease outcome (Matkowski et al. 2009) or no prognostic impact at all (Aaltomaa et al. 1992). High infiltration of CD8^+^ TILs has also been suggested to predict response to immune checkpoint blocking therapies (Rashidian et al. 2017). We did not detect significant prognostic impact for the other studied TIL subpopulations although in some malignancies improved survival has been detected in association with increased frequency of FoxP3^+^ or CD20^+^ lymphocytes (Mao et al. 2016).

According to evidence from gene expression profiling, immune response and proliferation are interrelated features in malignancy (Nagalla et al. 2013; Bianchini et al. 2010). CIN and aneuploid DNA content—common features of TNBC—have been reported in association with upregulation of genes mediating pro-inflammatory signals of the tumor microenvironment (Santaguida et al. 2017). On the subcellular level, aneuploidy is most commonly encountered as a result of missegregation at the spindle poles caused by defects at the metaphase–anaphase transition (Haschka et al. 2018). Regulation of the metaphase–anaphase transition is considered one of the events during the cell cycle where the cell is at its most vulnerable and susceptible to genetic disorders (Dominguez-Brauer et al. 2015). The transition is critically regulated by the APC/C (Anaphase-Promoting Complex/or Cyclosome) involving Securin (Pituitary tumor-transforming gene 1 protein, PTTG1) and Separase (Extra spindle poles-like 1 protein, ESPI1) to drive the cell into chromosome segregation and anaphase progression (Musacchio 2015). In the present observations, immunohistochemically detected overexpression of Securin and Separase was associated with the area fraction of stromal TILs (*p* = 0.003 and *p* = 0.01, respectively) and overexpression of Securin with CD8^+^ TILs (*p* = 0.02). Also this finding insinuates that uncontrolled proliferation may be linked to inflammatory response in TNBC. In the literature, the benefits of immunotherapies in TNBC have been partly explained by the high mutational levels resulting in a large number of immunogenetic neoantigens (Brown et al. 2014). However, the exact mechanisms of the interaction between immune response and proliferation in cancer have not yet been thoroughly explained. However, accumulating data points at inflammatory mediators directly or indirectly downregulate DNA repair pathways and cell cycle checkpoints, thus destabilizing cancer cell genome and contributing to the accumulation of random genetic alterations (Hanahan and Weinberg 2011; Colotta et al. 2009).

In the literature, no univocal principles or clinically relevant guidelines can be found for quantifying the inflammatory response in malignancy. According to international recommendations for breast cancer (Salgado et al. 2015; Hendry et al. 2017; https://www.tilsinbreastcancer.org), we used full tumor sections to assess the area fractions of TILs while immunohistochemical identification of TIL subtypes was performed in TMA cores specifically chosen to represent tumor inflammation. Biopsy material was excluded from the study. The evaluations were performed in digitized images and, when applicable, using an image analysis software to standardize the quantifications. In the literature, contradictory perceptions reign on the impact of the localization of inflammation on the outcome of different malignancies, including TNBC (Li et al. 2019; Liu et al. 2012; Ali et al. 2014; Angell and Galon 2013; Galon et al. 2016). Based on our findings from different tumor compartments, the highest prognostic significances were observed for the area fraction of TILs in the center and the fraction of CD8^+^ TILs at the invasive front of the tumor. Previous literature also lacks systematic cutpoints applicable to classifying inflammatory response in malignancy. In agreement with the previous literature (Salgado et al. 2015), our analyses did not provide a single statistically significant cutpoint for the area fraction of TILs and, therefore, TIL was involved in the prognostic analyses as a continuous parameter. However, in statistical analyses supported by morphological observations, we were able to identify for CD8^+^ TILs a cutpoint which optimally distinguished patients alive vs dead of TNBC (≥ 14% vs < 14% CD8^+^ TILs, respectively). Obviously, this classification is not directly applicable to other patient materials or institutions. Taken together, the several sources of variation remain a challenge for the application of immune markers in routine clinical practice for patients suffering from TNBC (Denkert et al. 2016).

TNBC comprises 10–20% of all breast carcinomas and is characterized by aggressive behavior, young age at diagnosis and high risk of relapse and mortality. In addition, the challenges of TNBCs include lack of specific prognostic features and targeted therapies. Previous literature and the present results show that—despite being an established component of breast cancer staging—axillary lymph node status is not an independent prognostic feature in TNBC (Gangi et al. 2014). Instead, based on our material of a total of 179 patients with complete clinical data and up to 18-year follow-up, the area fraction of TILs and the frequency of CD8^+^ TILs comprised promising markers for survival in TNBC. The prognostic impact of these features of inflammatory response was also evident when combined with tumor size and postmenopausal status. Applications of inflammatory response in patient treatment may benefit from systematic principles and clinically relevant guidelines for evaluation. The results suggest that in future significant improvements in prognostication and treatment of TNBC may be reached by increased understanding of the cellular composition and interactions of the inflammatory tumor microenvironment.

## Data Availability

The datasets of the current study are available from Auria biobank, Turku University Hospital, Turku, Finland.
